# Lipocalin-2 Regulates Hippocampal Microglial Activation in Poststroke Depression

**DOI:** 10.3389/fnagi.2021.798335

**Published:** 2021-12-13

**Authors:** Li Wei, Yupeng Du, Yirui Xie, Xiaopeng Yu, Hui Chen, Yunqing Qiu

**Affiliations:** ^1^State Key Laboratory of Diagnostic and Treatment of Infectious Diseases, College of Medicine, The First Affiliated Hospital, Zhejiang University, Hangzhou, China; ^2^Department of Rehabilitation, The Third Affiliated Hospital of Zhejiang Chinese Medical University, Hangzhou, China; ^3^NHC Key Laboratory of Combined Multi-organ Transplantation, The First Affiliated Hospital, College of Medicine, Zhejiang University, Hangzhou, China

**Keywords:** poststroke depression (PSD), microglia, hippocampi, Lipocalin-2 (Lcn2), p38 mitogen-activated protein kinase (p38 MAPK)

## Abstract

**Background and Purpose:** Microglia play important role in poststroke depression (PSD), however, the exact mechanism was still unclear. The purpose of the study was to study the mechanism of microglial activation in PSD.

**Methods:** 24 rats were randomly divided into three groups: the PSD group (*n* = 10), the poststroke (PS) group (*n* = 7), and the sham group (*n* = 7). Primary hippocampal microglia were isolated and cultured, and recombined LCN2 protein was used to stimulate the cultured microglia. The protein expression of Iba1, P38 MAPK and PP38 MAPK was analyzed by western blotting; the LCN2 expression was measured by RT-qPCR, the serum LCN2 level and the NO level were analyzed by ELISA.

**Results:** Open field test scores (horizontal score, vertical score, and self-grooming score) and the serum LCN2 level were significantly decreased in the PSD group compared with the other two groups (*P* < 0.05). The serum LCN2 level was positively correlated with the horizontal score and negatively correlated with the self-grooming score in the open field test (*P* < 0.05). The relative protein level of Iba1 and the LCN2 mRNA level were significantly increased in the hippocampal region compared with other brain regions (*P* < 0.05), while the relative protein level of Iba1 and the LCN2 mRNA level were significantly increased in the PSD group compared with the other two groups (*P* < 0.05). The length, supernatant NO level, phagocytic ability and migration ability of LCN2-treated microglia were significantly increased compared with those of untreated microglia (*P* < 0.05). The relative protein levels of P38 MAPK and the PP38 MAPK significantly increased in hippocampal region in the PSD group and LCN2-treated hippocampal microglia (*P* < 0.05).

**Conclusion:** Hippocampal microglia are activated during PSD; LCN2 may regulate hippocampal microglial activation by the P38 MAPK pathway in the process of PSD.

## Introduction

Poststroke depression (PSD), one sequelae of stroke, severely affects the functional outcome of stroke survivors and is associated with high mortality (Robinson and Jorge, 2016). Approximately 33% of stroke survivors are affected by PSD, making it a serious social and public health problem worth investigating (Das and Rajanikant, 2018). The pathophysiology of PSD is presumably multifactorial, involving a combination of the pathogeneses of stroke and depression, but has not yet been clarified (Villa et al., 2018). This lack of understanding of the pathogenesis of PSD seriously affects the ability of clinicians to prevent and treat it.

Microglia are small macrophage-like glial cells that account for 10–15% of cells in the central nervous system (CNS) (Hansen et al., 2018). Microglia in the CNS are usually maintained in a quiescent state. When activated, they can perform many diverse functions that may be either beneficial or harmful depending on the situation (Boche et al., 2013). In response to various brain injuries, such as ischemic stroke, microglia are activated and polarized toward a proinflammatory phenotype or anti-inflammatory phenotype (Ma et al., 2017; Jiang et al., 2020). Depression has been described as a microglia-associated disorder, and in addition to excessive cell activation and an increase in cell number, a decrease in microglial number and microglial senescence have been observed in some depressed patients (Yirmiya et al., 2015), which means that when activated, the microglia number increased, following excessive cell activation, the activated microglia became death/apoptosis, then its number became decrease. However, whether the initial increase of number or the following number decrease were both closely correlated with microglia activation. Therefore, microglial activation is closely associated with PSD. However, the exact role and mechanism of microglial activation in PSD has not been clarified.

Lipocalin-2 (LCN2) is a member of the highly heterogeneous lipocalin family of secretory proteins (Bhusal et al., 2019). LCN2 has been implicated in the regulation of cell differentiation, apoptotic cell death, and cellular uptake of iron (Ferreira et al., 2015). LCN2 is released by injured neurons as a “help-me” distress signal that activates microglia and astrocytes into potentially pro-recovery phenotypes (Xing et al., 2014). Recent studies have indicated that LCN2 expression and secretion by glial cells are induced by inflammatory stimuli in the CNS (Nam et al., 2014). LCN2 amplifies the M1 polarization of activated microglia (Jang et al., 2013). LCN2 is crucial for the effects of PRX-2 on neutrophil infiltration and microglia/macrophage activation (Zhang et al., 2021). All of the above findings indicate that LCN2 may play an important role in microglial activation.

Moreover, LCN2 has been reported to be highly expressed following ischemic stroke (Falke et al., 2000; Deng et al., 2019) and to be closely associated with major depressive disorder (Noh et al., 2019). Among proteins, LCN2 shows the most substantial elevation in expression in the CNS after peripheral administration of lipopolysaccharide (LPS) (Kang et al., 2018). Therefore, LCN2 is closely associated with PSD.

Microglial activation plays an important role in PSD, meanwhile LCN2 is closely associated with microglial activation; nonetheless, the exact role and mechanism of LCN2 in microglia activation in PSD are still unclear.

The present study aimed to determine whether and how LCN2 regulates microglial activation in PSD, potentially providing new insight into and a reference for the mechanism of PSD.

## Materials and Methods

### Poststroke Depression Animal Model Establishment

**FIGURE 1:**
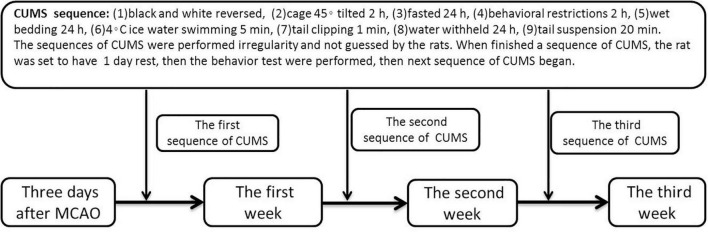
Chart of CUMS.

A total of 24 male Wistar rats (body weight, 200–250 g, 6–8weeks) were purchased from the Zhejiang Experimental Animal Center (Hangzhou, China). Considering the convenience of the operation and to eliminate the interference of estrogen of female rats, 6–8weeks male Wistar rats were selected in this study. The rats were randomly divided into three groups by random number method: the poststroke (PS) group (*n* = 7), the PSD group (*n* = 10), and the sham group (*n* = 7). The rats in the PS group were subjected to 90 min of middle cerebral artery occlusion (MCAO) achieved by common carotid artery and external carotid artery ligation with a nylon monofilament suture coated with silicone (403556PK5Re, Doccol) and reperfusion under chloral hydrate anesthesia (300 mg/kg, i.p.). After the procedure, the monofilament suture was removed, and the wound was sutured. The rats in the PSD group were subjected to the same MCAO operation as those in the PS group. Three days after MCAO, the rats were individually housed and subjected to 21 days of chronic unpredictable mild stress (CUMS) according to a previously reported method (Hu et al., 2019) (which exactly showed in Figure 1). The rats in the sham group were subjected to skin incision and anesthesia only (one rat died on day 4 post modeling). All procedures were carried out under strict compliance with the ethical principles and guidelines of the Chinese Council on Animal Care. All treatment and testing procedures were approved by the Animal Care Committee of the First Affiliated Hospital, College of Medicine, Zhejiang University.

The rats in the three groups were regularly subjected to a behavioral test (the open field test) during the experiments (1, 2, and 3 weeks postoperation).

Peripheral blood was obtained from the rats in the three groups during the experiments (1, 2, and 3 weeks postoperation). The serum was allowed to clot in a serum separator tube (for approximately 4 h) at room temperature and centrifuged at approximately 1,000 × g for 15 min. Then, the serum was immediately stored at –80°C until analysis.

### Microglial Cell Isolation and Culture

Primary microglial cultures were prepared as described previously with slight modifications (Tamashiro et al., 2012). Briefly, primary microglia were obtained from the hippocampi of newborn Sprague-Dawley rats. The Sprague-Dawley rats (1–3 days old) were obtained from the Zhejiang Experimental Animal Center (Hangzhou, China); all procedures were carried out under strict compliance with the ethical principles and guidelines of the Chinese Council on Animal Care. All treatment and testing procedures were approved by the Animal Care Committee of the First Affiliated Hospital, College of Medicine, Zhejiang University. The dissected hippocampi were collected and digested with digestion solution containing 0.25% trypsin-EDTA + 0.1% DNase I (Gibco) at 37°C for 30 min and then mechanically triturated using a 1 ml pipette tip. After 5 min, the trypsin was deactivated with DMEM (Gibco, United States) containing 10% FBS (Gibco) and 1% penicillin/streptomycin (Sigma-Aldrich, Billerica, MA, United States). The digested tissues were mechanically dissociated into single-cell suspensions, and the cells were collected by centrifugation. The cells were then suspended in DMEM containing 10% FBS and plated in T75 cell culture dishes [precoated with 20 μg/mL poly-D-lysine (Sigma-Aldrich)] at a density of 10^6^ cells/dish. After 12–14 days, the supernatant containing microglia was collected and centrifuged, and the cells were suspended at the appropriate density depending on the experiment. Microglia were identified by immunocytochemical staining using a microglia-specific antibody (a mouse anti-CD11b + CD11c antibody [OX42] (Alexa Fluor^®^ 647-conjugated), 1:100, ab216524, Abcam). The morphology of the isolated microglia was examined by phase-contrast microscopy (Nikon), and a confocal microscope (Zeiss, LSM 710, Germany) was used to capture the fluorescence images.

### Immunofluorescence Staining

Cultured microglia were fixed in 4% paraformaldehyde for 30 min and permeabilized with 0.3% Triton X-100 diluted in phosphate-buffered saline (PBS) for 5 min at room temperature. Then, the microglia were blocked with 2% bovine serum albumin in PBS for 1 h at room temperature and incubated with a mouse anti-CD11b + CD11c antibody [OX42] (Alexa Fluor^®^ 647-conjugated) (1:100, ab216524, Abcam) at 4°C overnight. Secondary antibodies were applied for 1.5 h at room temperature. The nuclei were stained with 4′,6–diamidino-2-phenylindole (DAPI). Images were analyzed by confocal microscopy (Zeiss, LSM 710, Germany) (Supplementary Material).

### Activation of Microglia by Recombinant Lipocalin-2 Protein

Harvested microglia were seeded at a density of 10^6^ cells/well in a six-well culture plate precoated with poly-D-lysine. 24 h after seeding, each well was washed three times with 0.1 M PBS, and the cells were incubated in culture medium containing 1 μg/mL recombinant LCN2 protein (ab108563, Abcam) for 24 h.

### Microglial Phagocytosis Assay

Primary microglia [untreated microglia (Con-MG) and microglia treated with recombinant LCN2 protein for 24 h (LCN2-MG)] were incubated with 3 μL of a 1:10 dilution of 1-mm Fluospheres (carboxylate-modified microspheres, F8821, Invitrogen, United States) for 2 h at 37°C (in humidified air containing 5% CO_2_ and 95%). The cells were then washed three times with ice-cold PBS and fixed in 4% paraformaldehyde for 30 min at room temperature. The nuclei were stained with DAPI. The number of beads per cell was counted. More than 60 cells per condition were analyzed by confocal microscopy (Zeiss, LSM 710, Germany) in each experiment, and at least three independent experiments were performed.

### Microglial Migration Assay

Freshly collected microglia treated with or without LCN2 for 24 h were seeded on collagen-coated Transwell membrane inserts (Corning Co., Corning, NY, United States), which were placed in the wells of a 12-well plate. The pores of the Transwell membranes, where were 3.0 μm, prevented direct cell-cell interactions but allowed diffusion of soluble factors across the membrane. After 24 h, the cultured cells were separated and cultured in fresh culture medium for another 24 h, and then the supernatant and cells were collected for analysis. The cells were divided into two groups: the Con-MG and LCN2-MG groups.

### ELISA

The serum LCN2 level was measured with a commercial LCN2 rat ELISA kit (ab119602, Abcam) according to the manufacturer’s instructions.

The concentration of NO in the supernatant of cultured microglia was measured with a commercial NO rat ELISA kit (S0023, Beyotime, China) according to the manufacturer’s instructions.

### RNA Isolation and Real-Time PCR

RNA was extracted from brain tissue with TRIzol™ reagent (Invitrogen™, United States). Then, 2 μg of each RNA sample was converted to cDNA using a Reverse Transcription System (Promega, United States) according to the manufacturer’s instructions. Real-time PCR was performed using TB Green^®^ Premix Ex Taq™ II (Tli RNaseH Plus) (Takara Bio Inc., Tokyo) and an ABI Prism R 7,500 sequence detection system according to the manufacturer’s instructions. The primer sequences were: β-actin (forward) 5′-CGTTGACATCCGTAAAGACCTC-3′ and (reverse) 5′TAGGAGCCAGGGCAGTAATCT-3′; LCN2 (forward) 5′-AACGTCACTTCCATCCTCGTC-3′ and (reverse) 5′-AATCGCTCCTTCAGTTCATCG-3′. The thermal cycling conditions were 95°C for 30 s followed by 40 cycles of 95°C for 5 s and 60°C for 34 s. Melting curve profiles were generated at the end of each reaction. The mRNA expression level of LCN2 was normalized to that of β-actin, and the 2^–△^^△^^Ct^ method was used for analysis.

### Western Blot Analysis

Twenty-four hours following completion of the behavioral test, the rats were anaesthetized with chloral hydrate (300 mg/kg, i.p.) and decapitated, and hippocampal tissues were carefully dissected and lysed on ice for 30 min in RIPA buffer containing a cocktail of protease/phosphatase inhibitors. The protein concentration was determined using a BCA protein assay kit (BioTek Epoch, US). A total of 30 μg protein from each tissue sample was loaded in each lane of an SDS-PAGE gel, and the proteins were electrophoretically separated and then transferred onto PVDF membranes, which were incubated with primary antibody [rabbit anti-Iba1 (ab178847, Abcam), p38 mitogen-activated protein kinase (P38 MAPK; D13E1) XP^®^ rabbit mAb (8,690, Cell Signaling Technology), and phospho-p38 MAPK (PP38 MAPK) (Thr180/Tyr182) (D3F9) XP^®^ rabbit mAb (4,511, Cell Signaling Technology)] at 4°C overnight. The secondary antibody was a horseradish peroxidase (HRP)-conjugated antibody (1:5,000, goat anti-rabbit IgG H&L, ab205718, Abcam). The blots were developed using an enhanced chemiluminescence detection kit (GE Healthcare, Buckinghamshire, United Kingdom), and the protein band densities were quantified using ImageJ software (NIH, Scion Corporation, Frederick, MD).

The cultured microglia were washed two times with PBS and collected in RIPA buffer containing a cocktail of protease/phosphatase inhibitors. Then western blot analysis was performed in the same manner as for hippocampal tissue.

### Statistical Analysis

All data are presented as the mean ± standard deviation (SD). SPSS 20.0 software and GraphPad Prism 5 software were used to perform the statistical analysis. Descriptive statistics were used to determine whether data were normally distributed. Comparisons between three groups were performed with one-way analysis of variance (ANOVA), and a *post hoc* test was used to identify differences between groups. Comparisons between two groups were performed with independent sample *t*-tests. Pearson correlation analysis was performed to assess associations between serum LCN2 levels and open field test scores. A *p*-value less than 0.05 was considered statistically significant.

## Results

### Decreased Open Field Test Scores in the Poststroke Depression Group

**FIGURE 2:**
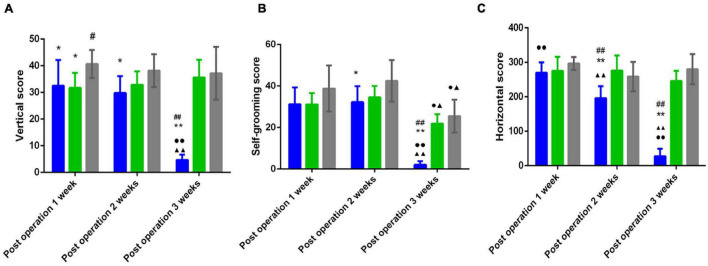
**(A)** Vertical scores in the open field test of three groups. **(B)** Self grooming scores in the open field test of three groups. **(C)** Horizontal scores in the open field test of three groups. [^#^Compare with the PS group < 0.05, ^##^Compare with the PS group, *P* < 0.01; *Compare with the Sham group, *P* < 0.05, **Compare with the Sham group < 0.01; ^▲^Compare with post operation 1 week, *P* < 0.05, ^▲▲^Compare with post operation 1 week, *P* < 0.01; ^∙^Compare with post operation 2 weeks, *P* < 0.05, ^∙∙^Compare with post operation 2 weeks, *P* < 0.01; Sham group (*n* = 6), PS group (*n* = 7), and PSD group (*n* = 10)].

Figure 2 shows that compared with those of the sham group, the horizontal score, the vertical score and the self-grooming score of the PSD group were significantly decreased 2 weeks and 3 weeks postoperation (*P* < 0.05); compared with that of the PS group, the horizontal score of the PSD group was significantly decreased 2 weeks and 3 weeks postoperation (*P* < 0.01), and the vertical score and the self-grooming score of the PSD group was significantly decreased 3 weeks postoperation (*P* < 0.05, Figure 2).

### Sustained Elevation of Serum Lipocalin-2 Concentration in the Poststroke Depression Group at 1, 2, and 3 Weeks Postoperation

**FIGURE 3:**
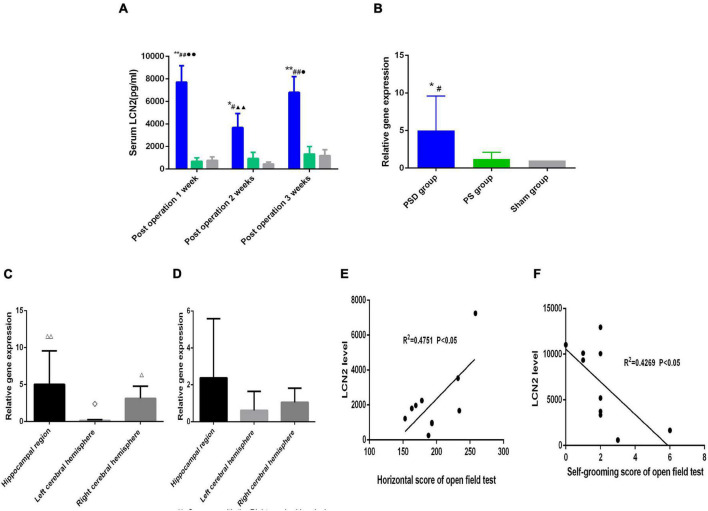
**(A)** Serum LCN2 level in the three groups. **(B)** Relative LCN2 mRNA levels in the hippocampal region in the three groups. **(C)** Relative LCN2 mRNA levels in different brain regions in the PSD group. **(D)** Relative LCN2 mRNA levels in different brain regions in the PS group. **(E)** Correlation of serum LCN2 level with the horizontal score of open field 2 weeks postoperation. **(F)** Correlation of serum LCN2 level with the self-grooming score of open field 3 weeks post operation. [^#^Compare with the PS group, *P* < 0.05, ^##^Compare with the PS group, *P* < 0.01; *Compare with the Sham group, *P* < 0.05, **Compare with the Sham group, *P* < 0.01; ^▲▲^Compare with post operation 1 week, *P* < 0.01; ^∙^Compare with post operation 2 weeks, *P* < 0.05, ^∙∙^Compare with post operation 2 weeks, *P* < 0.01; ^⋄^Compare with the right cerebral hemisphere, *P* < 0.05; ^△^ Compare with the left cerebral hemisphere, *P* < 0.05, ^△^^△^ Compare with the Left cerebral Hemisphere, *P* < 0.01; Sham group (*n* = 6), PS group (*n* = 7), and PSD group (*n* = 10)].

Figure 3A shows that compared with that in the sham group, the concentration of serum LCN2 in the PSD group was significantly increased 1 week, 2 weeks, and 3 weeks postoperation (*P* < 0.05). Compared with that in the PS group, the concentration of serum LCN2 in the PSD group was significantly increased 1 week, 2 weeks, and 3 weeks postoperation (*P* < 0.05). The concentration of serum LCN2 in the PSD group was significantly decreased 2 weeks postoperation compared with 1 week postoperation (*P* < 0.05). The concentration of serum LCN2 in the PSD group was significantly increased 3 weeks postoperation compared with 2 weeks postoperation (*P* < 0.05, Figure 3A).

### Sustained Elevation of Lipocalin-2 mRNA Expression in the Hippocampal Region in the Poststroke Depression Group

Figure 3B shows that compared with that the sham group and the PS group, the relative mRNA expression of LCN2 was significantly increased in the PSD group (*P* < 0.05, Figure 3B). Figure 3C shows that in the PSD group, the relative mRNA expression of LCN2 was significantly decreased in the left cerebral hemisphere compared with the right cerebral hemisphere (*P* < 0.05) and that the relative mRNA expression of LCN2 was significantly increased in the hippocampal region and right cerebral hemisphere compared with the left cerebral hemisphere (*P* < 0.01, Figure 3C). Figure 3D shows that in the PS group, there was no significant difference in the relative mRNA expression of LCN2 between different brain regions (*P* > 0.05, Figure 3D).

### The Serum Lipocalin-2 Level Was Positively Correlated With the Horizontal Score and Negatively Correlated With the Self-Grooming Score

Figure 3E showed that the serum LCN2 level was positively correlated with the horizontal score (*R*^2^ = 0.4751, *P* < 0.05, Figure 3E) 2 weeks postoperation, and that the serum LCN2 level was positively correlated with the self-grooming score (*R*^2^ = 0.4269, *P* < 0.05, Figure 3F) 3 weeks postoperation.

### Increased Iba1 Protein Levels in the Hippocampal Region Compared With Other Regions in Poststroke Depression Rats

**FIGURE 4:**
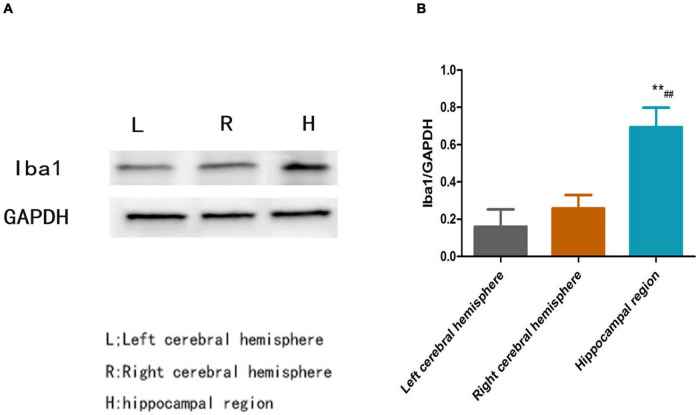
**(A)** Blots of Iba1 in different brain regions in the PSD group. **(B)** Relative protein level of Iba1 in different brain regions in the PSD group [**Compare with the left cerebral hemisphere, *P* < 0.01; ^##^Compare with the right cerebral hemisphere, *P* < 0.01; Left cerebral hemisphere (*n* = 3), Right cerebral hemisphere (*n* = 3), Hippocampal region (*n* = 3)].

Figure 4 shows that the relative protein level of Iba1 was obviously increased in the hippocampal region compared with the left cerebral hemisphere and the right cerebral hemisphere in PSD rats (*P* < 0.01, Figure 4B); however, there was no significant difference in Iba1 protein expression between the left cerebral hemisphere and the right cerebral hemisphere (*P* > 0.05, Figure 4).

### Increased Iba1, P38 MAPK Protein Levels and the Ratio of PP 38/P38 in the Hippocampal Region in Poststroke Depression Rats

**FIGURE 5:**
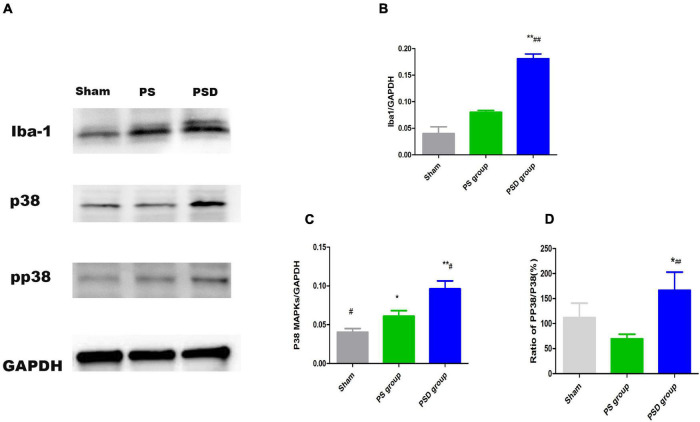
**(A)** Blots of Iba1, P38 MAPK, and PP38 MAPK in the three groups. **(B)** Relative protein levels of Iba1. **(C)** Relative protein levels of P38 MAPK. **(D)** Ratio of PP38/P38 MAPK [^#^Compare with the PS group < 0.05, ^##^Compare with the PS group, *P* < 0.01; *Compare with the Sham group, *P* < 0.05, **Compare with the Sham group, *P* < 0.01; Sham group (*n* = 3), PS group (*n* = 3), and PSD group (*n* = 3)].

Figure 5 shows that the relative protein levels of Iba1, P38 MAPK and PP 38 MAPK in the hippocampal region were obviously increased in the PSD group compared with those in the sham and PS groups (*P* < 0.01). Compared with those in the PS group, the relative protein levels of Iba1, P38 MAPK protein levels and the ratio of pP38/P38MAPK in the PSD group were significantly increased (*P* < 0.05, Figures 5B–D).

### Alterations in Microglia After Lipocalin-2 Stimulation

**FIGURE 6:**
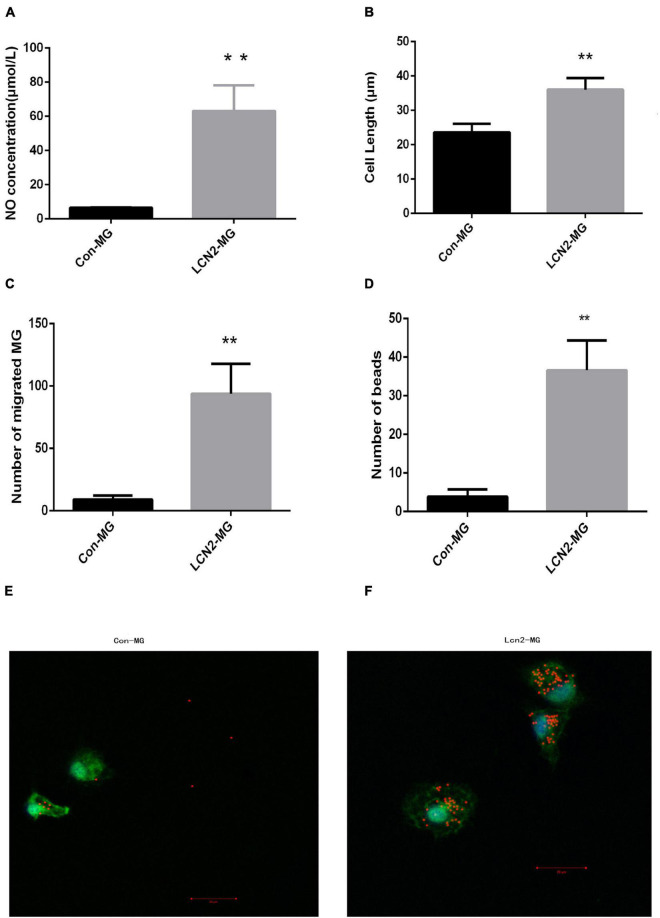
**(A)** NO level in the supernatant of primary microglia. **(B)** Length of primary microglia. **(C)** Migration ability of primary microglia. **(D)** Phagocytic ability of primary microglia. **(E)** Uptake of 1 ml microspheres for 5 h by Con-MG. **(F)** Uptake of 1 ml microspheres for 5 h by LCN2-MG. (Primary microglia were identified by Iba1 expression (green), and nuclei were stained with DAPI (blue). Scale bars: 20 μm. [**Compare with Con-MG, *P* < 0.01. Con-MG (*n* = 3), LCN2-MG (*n* = 3)].

Figure 6A shows that after treatment with 1 μg/ml LCN2 for 24 h, the concentration of NO in supernatant of LCN2-MG was significantly increased compared with that in the supernatant of Con-MG (*P* < 0.01, Figure 6A). LCN2-MG were significantly longer than Con-MG (P < 0.01, Figure 6B), and more LCN2-MG than Con-MG migrated (*P* < 0.01, Figure 6C). The number of microspheres phagocytosed by LCN2-MG was significantly higher than that phagocytosed by Con-MG (*P* < 0.01, Figure 6D). Figures 6E,F show that LCN2 enhanced the phagocytic ability of primary microglia.

### Increased Iba1, P38 MAPK and PP38 MAPK Protein Levels in Lipocalin-2-Treated Microglia

**FIGURE 7:**
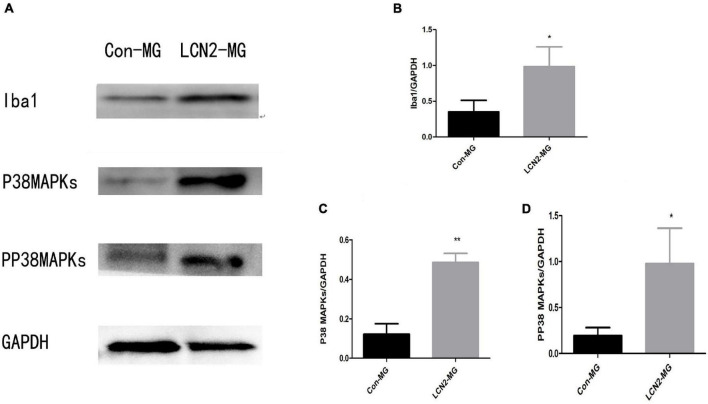
**(A)** Blots of Iba1, P38 MAPK, and PP38 MAPK in primary microglia. **(B)** Relative protein levels of Iba1 in primary microglia. **(C)** Relative protein levels of P38 MAPK Iba1 in primary microglia. **(D)** Relative protein levels of PP38 MAPK in primary microglia [*Compare with Con-MG, *P* < 0.05, **Compare with Con-MG, *P* < 0.01.Con-MG (*n* = 3), LCN2-MG (*n* = 3)].

Figure 7 shows that the relative protein levels of Iba1, P38 MAPK and PP38 MAPK were significantly increased in LCN2-MG compared with Con-MG (*P* < 0.05, Figures 7B–D).

## Conclusion

In summary, in this study, we found that LCN2 may regulate hippocampal microglial activation through the P38 MAPK pathway in PSD.

In our previous paper, we used Despair swimming test (DST) and Sucrose preference test (SPT) to test the depression behavior of PSD rat, and measured the rate of weight change of the rats, our PSD model rats exhibited multiple depressive symptoms (Wei et al., 2021). And in this study we performed another behavior test: open field test, and found that open field test scores (the horizontal score, vertical score, and self-grooming score) were significantly decreased in the PSD group compared with the other two groups. The serum LCN2 level was obviously increased in the PSD group compared with the other two groups. Correlation analysis showed that the serum LCN2 level was positively correlated with the horizontal score and negatively correlated with the self-grooming score in the open field test. Furthermore, we found that the mRNA level of LCN2 was significantly increased in the hippocampi of PSD rats and that the LCN2 mRNA level was significantly increased in the hippocampus compared with other brain regions. These results indicated that LCN2 is closely associated with PSD.

The hippocampus is involved in many essential functions, particularly learning, memory, emotions, adaptation, and neuronal generation. Pathological alterations in the hippocampus underlie various mental and neurological diseases, particularly depression, dementia, and epilepsy (Gulyaeva, 2019). Microglial activation is closely associated with PSD (Yirmiya et al., 2015; Ma et al., 2017; Jiang et al., 2020). Therefore, studying hippocampal microglial activation is important for clarifying the mechanism of PSD. In our previous study we analyzed the microglia activation markers in the hippocampal region, the right cerebral and the left cerebral hemispheres in the PSD rat, and found that hippocampus showed a more microglial activation status than the other brain regions (Wei et al., 2021), meanwhile in our study we found that the protein levels of the microglial activation marker Iba1 were significantly increased in the hippocampal region in PSD rats. Our results indicated that hippocampal microglia were activated in PSD. Therefore we focus hippocampus to study.

LCN2 was shown to be closely associated with PSD, and hippocampal microglia were shown to be activated in PSD. However, the exact relationship between LCN2 and hippocampal microglial activation in PSD is still unclear. To clarify the relationship between LCN2 with hippocampal microglial activation, we cultured primary hippocampal microglia and stimulated them with recombinant LCN2 protein. We found that after LCN2 stimulation, primary microglia showed significant alterations: the amount of NO released by microglia, length of the microglia, and phagocytic ability and migration ability of hippocampal microglia significantly increased. Our results showed that LCN2 may regulate hippocampal microglial activation. However, the exact pathway by which LCN2 regulates hippocampal activation has not been clarified.

Microglial activation has been reported to be regulated by proinflammatory factors (Liu et al., 2018). Activation of p38 MAPK in spinal microglia results in increased synthesis and release of the neurotrophin brain-derived neurotrophic factor and the proinflammatory cytokines interleukin-1β, interleukin-6, and tumor necrosis factor-α (Wen et al., 2011). *In vivo* evidence suggests that P38 MAPK may play a critical role in detrimental microglial activation following acute brain injury, such as stroke, and in more chronic neurodegenerative diseases, such as Alzheimer’s disease (Koistinaho and Koistinaho, 2002).

Due to the close relationships of P38 MAPK and LCN2 with microglial activation, we speculate that LCN2 may induce microglial activation through P38 MAPK. Therefore, we assessed alterations in P38 MAPK protein levels in hippocampal microglia after LCN2 stimulation and found that the relative protein level of P38 MAPK significantly increased. Moreover, the PP38 MAPK protein level showed the same significant increase, and the increase in the PP38 MAPK protein level indicated that P38 PAPK was activated (Martinez-Limon et al., 2020). Our results indicated that LCN2 may induce hippocampal microglial activation through activation of the P38 MAPK pathway. P38 MAPK and PP38 MAPK protein levels showed the same changes in the hippocampi of PSD rats as in cultured primary microglia. The *in vitro* and *in vivo* results indicated that LCN2 may regulate hippocampal microglial activation through the P38 MAPK pathway in PSD.

The limitations and shortcomings of our study were that we studied the whole hippocampus but not different functional areas of the hippocampus. Further analysis of microglial activation in different functional areas of the hippocampus is needed.

In summary, we found that LCN2 may regulate hippocampal microglial activation through the P38 MAPK pathway in PSD. Our results may provide new insight for clarifying the mechanism of PSD.

## Data Availability Statement

The original contributions presented in the study are included in the article/Supplementary Material, further inquiries can be directed to the corresponding author/s.

## Ethics Statement

The animal study was reviewed and approved by Animal Care Committee of the First Affiliated Hospital, College of Medicine, Zhejiang University.

## Author Contributions

LW, YD, and YX carried out the PSD model establishing and primary microglia culture participated in the design of the study and helped to draft the manuscript. XY and HC participated in the RT-PCR and western blot analysis. YQ conceived the study, participated in its design and coordination, and helped to draft the manuscript. All authors read and approved the final manuscript.

## Conflict of Interest

The authors declare that the research was conducted in the absence of any commercial or financial relationships that could be construed as a potential conflict of interest.

## Publisher’s Note

All claims expressed in this article are solely those of the authors and do not necessarily represent those of their affiliated organizations, or those of the publisher, the editors and the reviewers. Any product that may be evaluated in this article, or claim that may be made by its manufacturer, is not guaranteed or endorsed by the publisher.

## Abbreviations

PSD: Poststroke depression
LCN2: Lipocalin-2
P38 MAPK: P38 mitogen-activated protein kinase
CNS: central nervous system
MCAO: middle cerebral artery occlusion
CUMS: chronic unpredictable mild stress
LPS: lipopolysaccharide.
