# Supplementary figures and images for: Lipocalin-2 Regulates Hippocampal Microglial Activation in Poststroke Depression
Source: Front Aging Neurosci. 2021 Dec 13;13:798335. doi: 10.3389/fnagi.2021.798335 (PMC8710735; doi:10.3389/fnagi.2021.798335)

# Immunofluorescence Staining of cultured microglia

**OX42-Red**

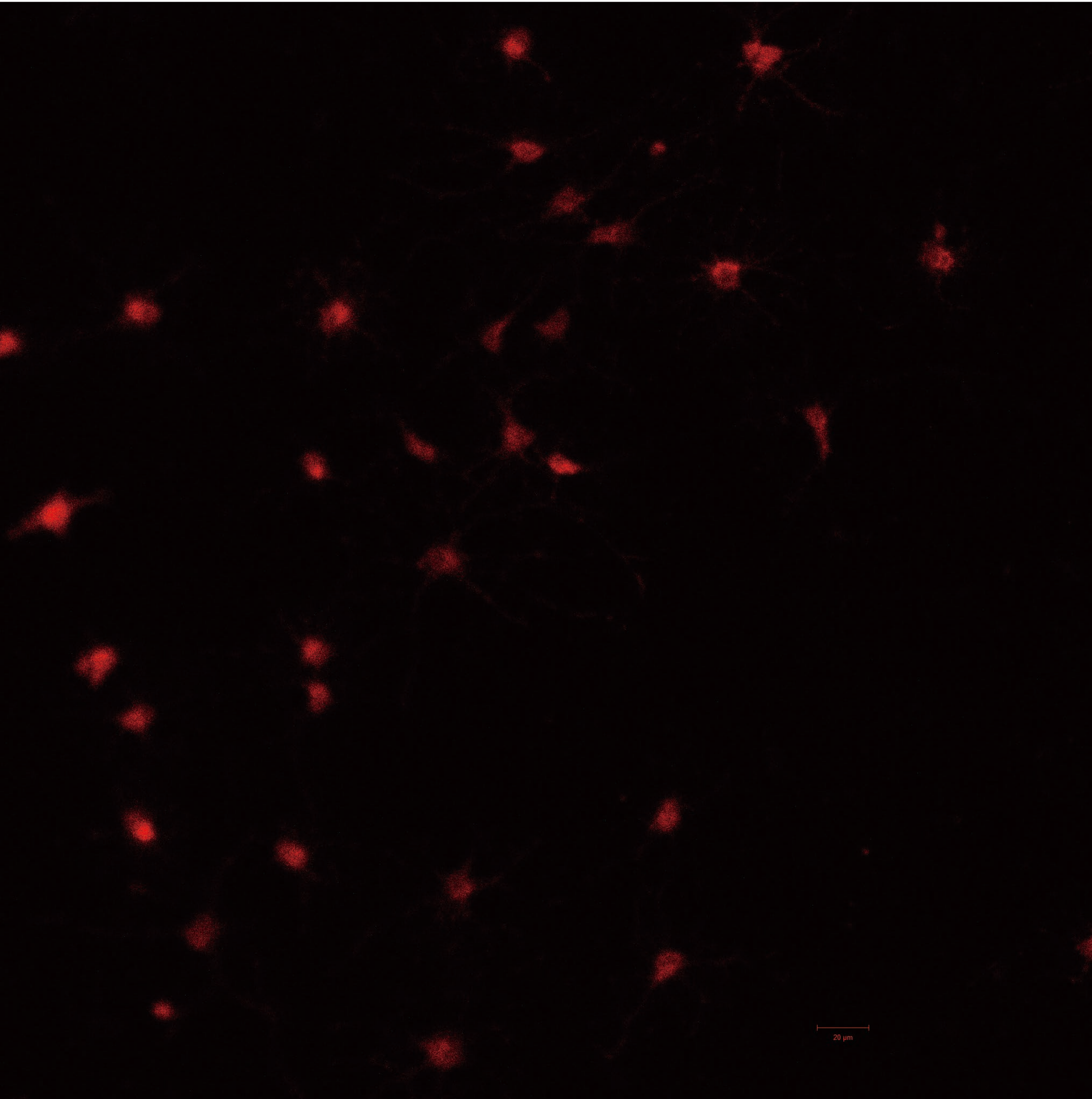

**DAPI-Blue**

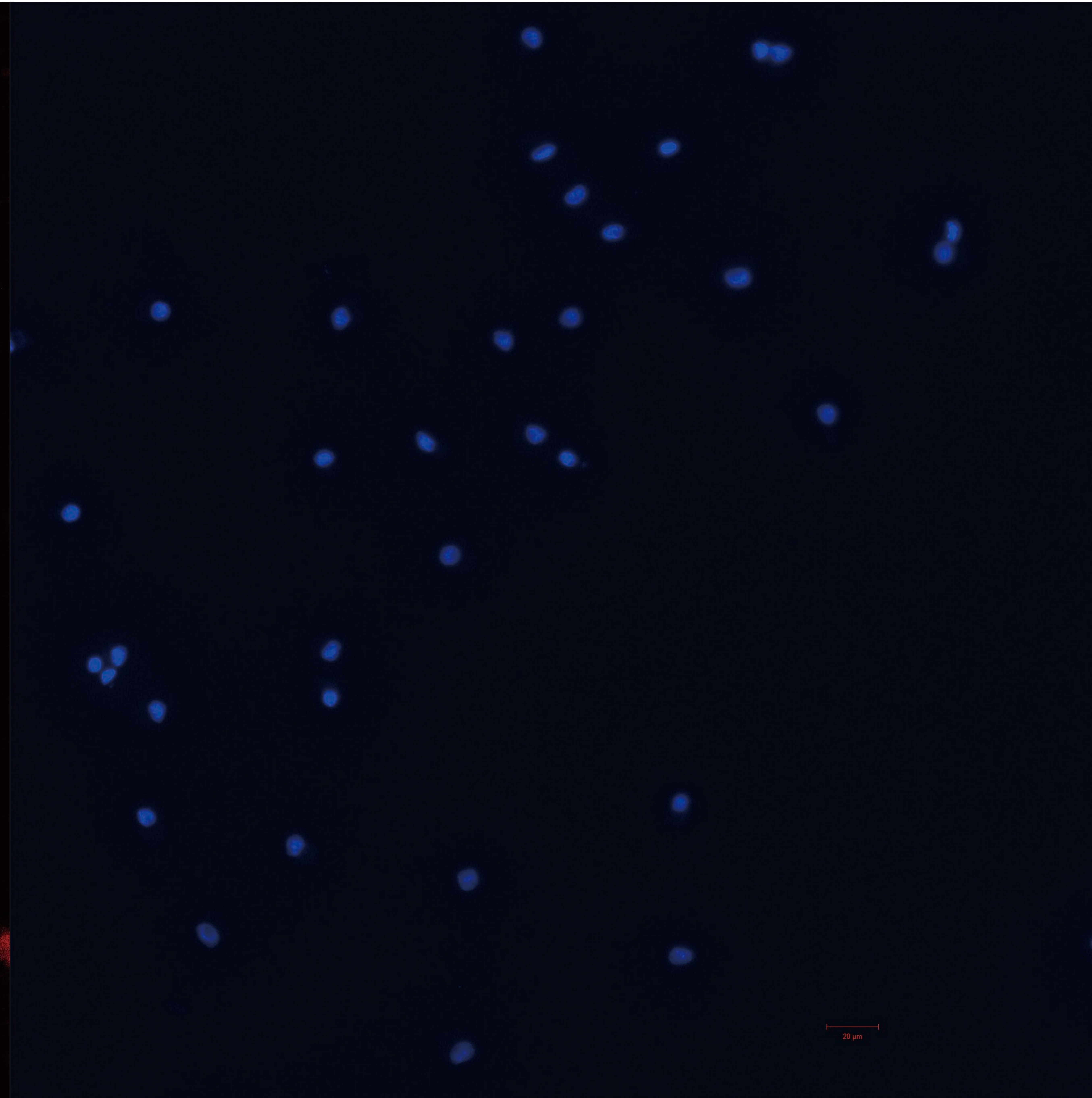

**Merge**

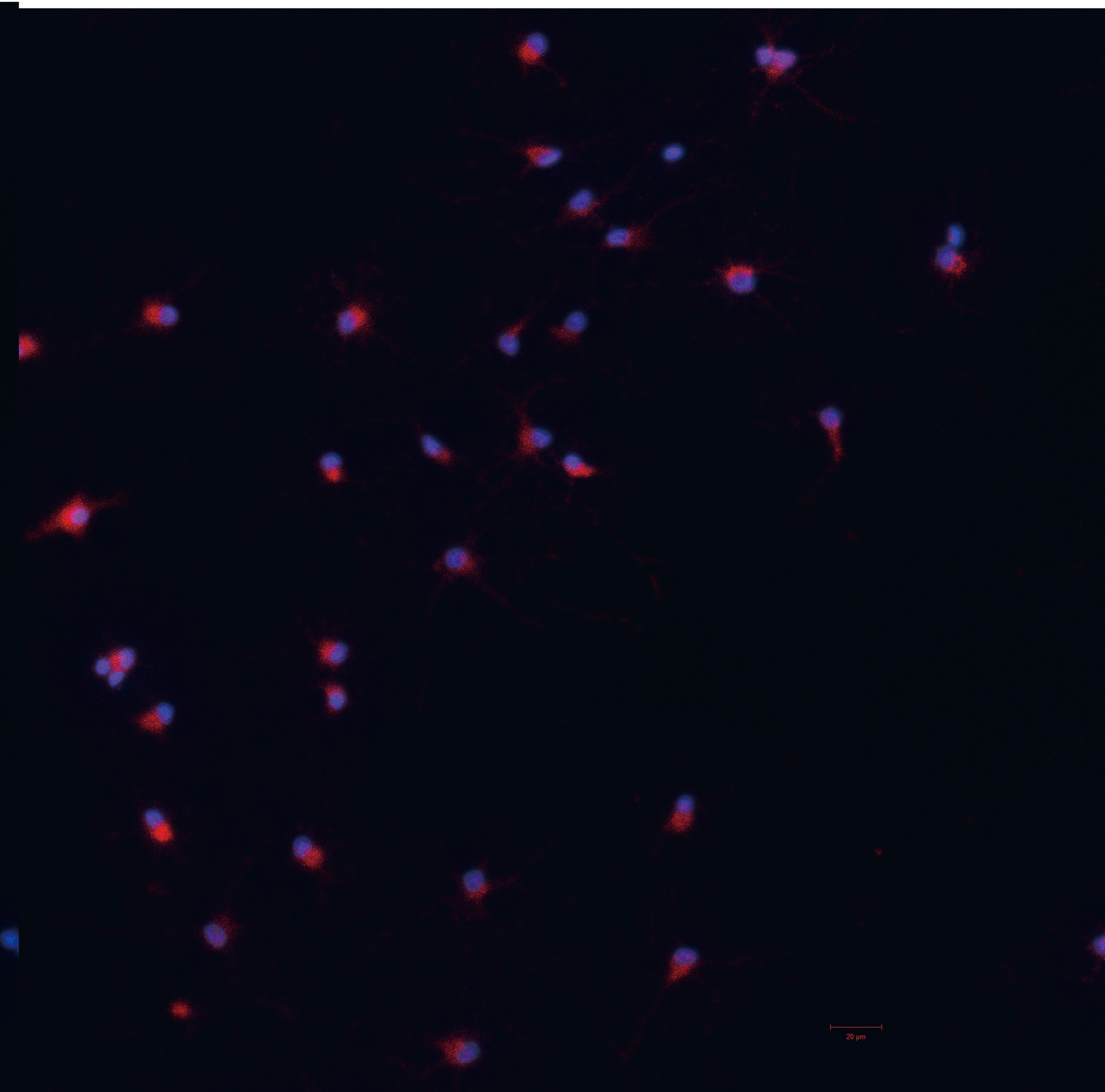

Supplement: Supplementary file 1 [file Data_Sheet_1.pdf]
